# QTL Mapping of Leafy Heads by Genome Resequencing in the RIL Population of *Brassica rapa*

**DOI:** 10.1371/journal.pone.0076059

**Published:** 2013-10-28

**Authors:** Xiang Yu, Han Wang, Weili Zhong, Jinjuan Bai, Pinglin Liu, Yuke He

**Affiliations:** National Key Laboratory of Plant Molecular Genetics, Shanghai Institute of Plant Physiology and Ecology, Shanghai Institutes for Biological Sciences, Chinese Academy of Sciences, Shanghai, China; Pennsylvania State University, United States of America

## Abstract

Leaf heads of cabbage (*Brassica oleracea*), Chinese cabbage (*B. rapa*), and lettuce (*Lactuca sativa*) are important vegetables that supply mineral nutrients, crude fiber and vitamins in the human diet. Head size, head shape, head weight, and heading time contribute to yield and quality. In an attempt to investigate genetic basis of leafy head in Chinese cabbage (*B. rapa*), we took advantage of recent technical advances of genome resequencing to perform quantitative trait locus (QTL) mapping using 150 recombinant inbred lines (RILs) derived from the cross between heading and non-heading Chinese cabbage. The resequenced genomes of the parents uncovered more than 1 million SNPs. Genotyping of RILs using the high-quality SNPs assisted by Hidden Markov Model (HMM) generated a recombination map. The raw genetic map revealed some physical assembly error and missing fragments in the reference genome that reduced the quality of SNP genotyping. By deletion of the genetic markers in which recombination rates higher than 20%, we have obtained a high-quality genetic map with 2209 markers and detected 18 QTLs for 6 head traits, from which 3 candidate genes were selected. These QTLs provide the foundation for study of genetic basis of leafy heads and the other complex traits.

## Introduction

With the completion of the reference genome, the 1000 Genomes Project (human) and the 1001 Genomes Project (Arabidopsis) have been performed using second generation sequencing techniques [1]. The single nucleotide polymorphisms (SNP) identified within the human population have been widely used in genome-wide association studies (GWAS) for multiple human diseases [2], [3], and complex trait loci in Arabidopsis, maize, and rice have also been identified by GWAS [4]–[7]. In plants, the identification of trait loci by high-resolution linkage mapping utilizing SNPs as markers in biparental cross populations provides a powerful complementary strategy to GWAS in a natural population [8]–[10]. At the genome-wide sequencing level, the distinction between the GWAS study, based on linkage disequilibrum (LD), and linkage mapping vanishes [11]. Compared to GWAS, linkage mapping has less generation and offspring from a biparental cross to shuffle the genome into smaller fragments, while GWAS exploits all of the recombination events that have occurred in the evolutionary history, which produces much higher mapping resolution. However, GWAS has little power to detect associations in low frequency alleles and QTL with small effects. In addition to possessing the sensitivity to detect QTL with low LOD, another advantage of linkage mapping is that the population from biparental cross can be further used for map-based cloning of the exact genes.

In rice, the SNPs and insertion/deletion (InDel) information between two subspecies (*indica* and *japonica*) is the basis for linkage mapping of trait loci in their cross population. Linkage mapping by genome resequencing of recombinant inbred lines opens the approaches to genotyping strategies for more effective genetic mapping and genome analysis. This method has substantially improved the efficiency of marker collection by allowing the detection of numerous markers in population of plant species whose genomes have been completely and accurately sequenced. But, it remains complex and ineffective in the species whose genome sequences are incomplete or inaccurate.

The species *Brassica rapa* include many important vegetable and oilseed crops, which are diverse in shape, size, curvature and inclination angles of leaves [12], [13]. Heading Chinese cabbage (*B. rapa* ssp. *pekinensis*) undergoes through three stages: seedling, rosette and heading. The seedling leaves and rosette leaves of heading and nonheading Chinese cabbage (*B. rapa* ssp. *chinenesis*) function in photosynthesis and respiration in the same way as the leaves of the other major crops, whereas the head leaves of heading Chinese cabbage serve as storage organs that supply mineral nutrients, crude fiber and vitamins of vegetables in the human diet. Head traits such as weight, size, shape, and heading time are the important components of vegetable yield and quality. In *B. rapa*, genetic linkage maps of some traits have been constructed using different kinds of molecular markers. Based on the analysis of gene function in trait loci, some well-known candidate genes have been identified [14]–[16]. Compared to the sequencing-based genotyping and linkage mapping in rice, the *B. rapa* crop presents some challenges. The published genome sequence of *B. rapa* ssp. *pekinensis* cv Chiifu-401-42 is 256 Mb in length, while 40 thousand scaffolds of 27-Mb (∼10%) remain unable to be located in a physical map [17], [18].

In this study, we have taken advantages of technical development of linkage mapping by genome resequencing to generate a population of 150 recombinant inbred lines (RILs) and to optimize SNP genotyping and linkage mapping using the genome sequences of *B. rapa* ssp. *Pekinensis*. cv chiifu-401-42 as the reference sequence.

## Results

### Intensive variance in head traits across 150 RIL lines

**Figure 1:**
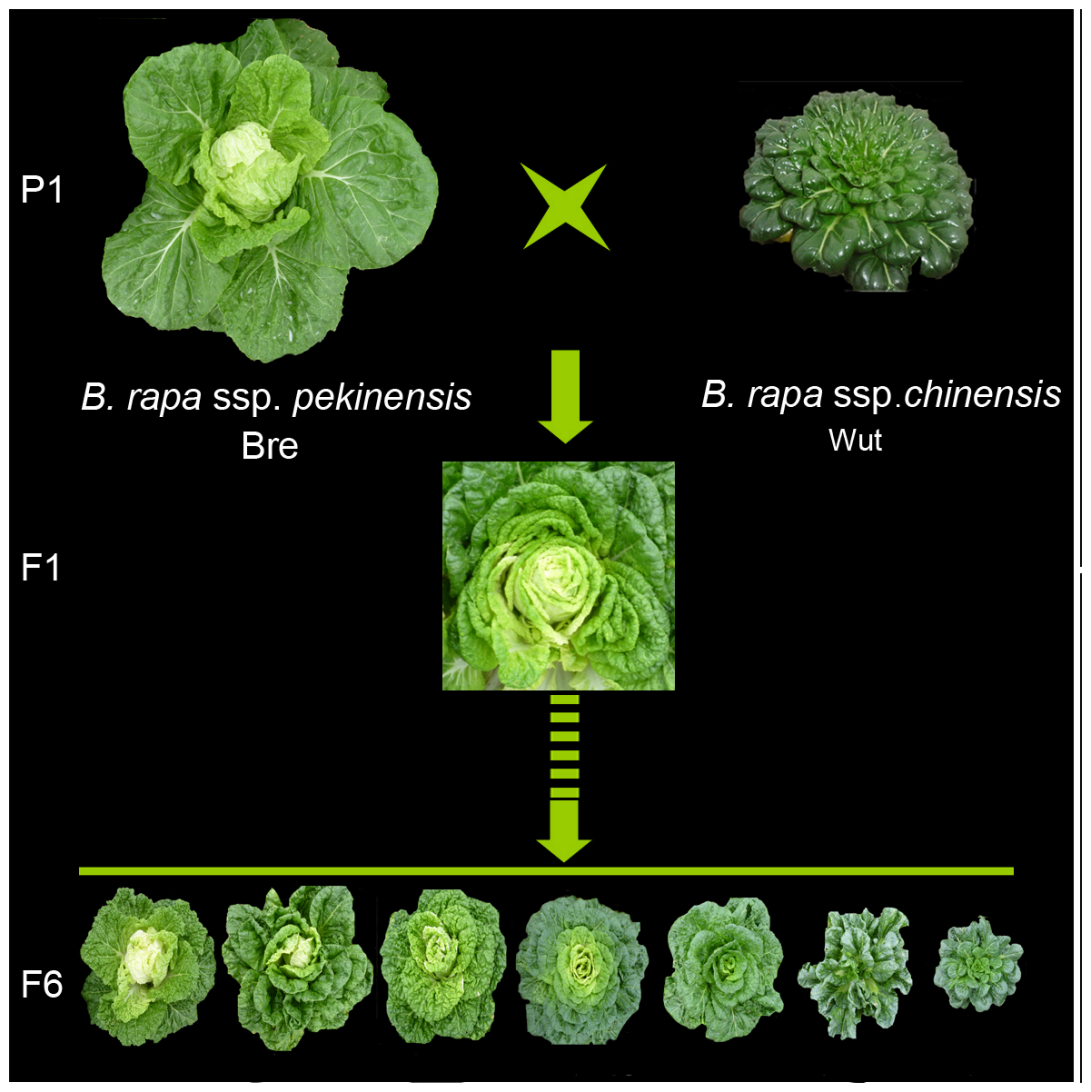
Leaf traits of heading Chinese cabbage and non-heading Chinese cabbage and their recombination lines.

The plants of Bre (heading Chinese cabbage) and Wut (non-heading Chinese cabbage) were distinct in phenotype (Fig. 1). Bre plants exhibited extremely large leaves with pale-green color and short petioles, compared with Wut plants. Noticeably, Bre leaves in the center of plants incurved and formed leafy heads, while Wut leaves curved downward, without formation of leafy heads. In order to investigate genetic basis of leafy heads and the related traits, we crossed Bre with Wut and performed successive self-fertilization of F1 progenies for 6 generations, and eventually generated a population of 150 recombinant inbred lines (RILs).

**Figure 2:**
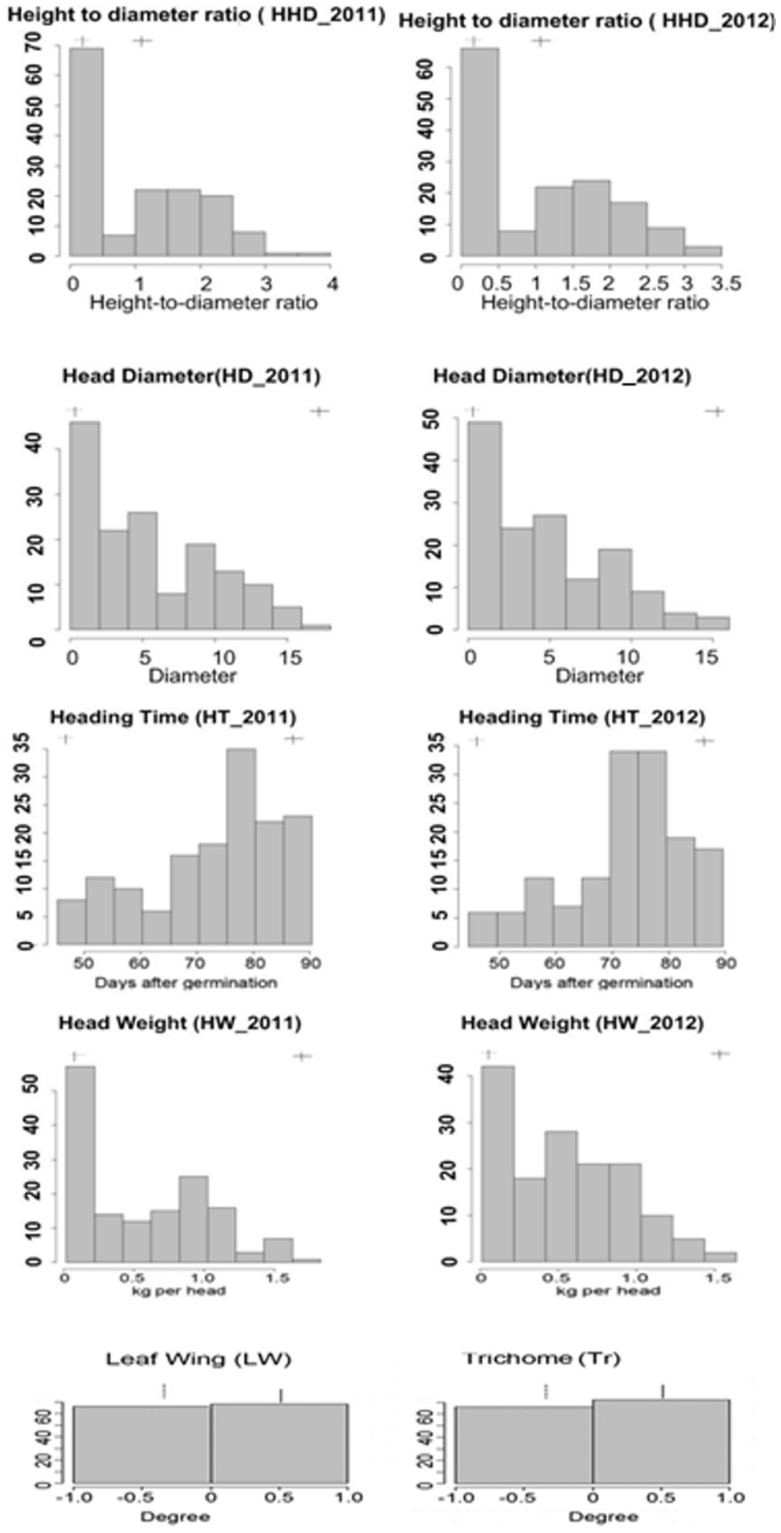
Frequency distributions of 6 head traits in the RIL lines and their parental lines. The vertical axis of each figure represents the number of RIL lines. Arrows show the mean values for the parental lines Bre and Wut.

The 150 RIL lines and the parental lines were grown in the field at the same seasons of 2011 and 2012. They exhibited intensive variance in plant phenotypes in two seasons. We measured head diameter (HD), head height-to-diameter ratio (HHD), head weight, and heading time at heading stage in 2011 and 2012, respectively (Table 1). The parameters of head traits between the two trails were strongly correlated (Table 2). We used the parameters recorded in 2011 and 2012 for the analysis of frequency distribution and QTL mapping. The frequency distribution of head traits was skewed (Fig. 2), due to the existence of a subset of plants without heads within the RIL population. Head leaves of Bre plants had leaf wings along petioles and trichome on abaxial sides while Wut leaves did not. The measurements of these two traits were the same in the two trails. Within the RIL population, number of RIL lines with trichome was almost the same as that observed without trichome, meaning that trichome on head leaves of Bre plants was a qualitative trait.

**Table 1:** Data of head traits in the parents and the RIL population.

Traits			2011				2012	
	P1	P2	RILs		P1	P2	RILs	
			Mean	Range			Mean	Range
HHD	0	1	1.1	0–4	0	1	1.13	0–3.5
HD	16.4	0.1	6.01	0.1–16.1	16.4	0.1	5.33	0.1–16.4
HT	45	75	6.98	45–90	55	90	7.02	45–90
HW	1.7	0	0.56	0–1.8	1.7	0	0.57	0–1.7

**Table 2:** The correlation coefficients between different traits and years.

Traits and Trials	HHD 2011	HD 2011	HT 2011	HW 2011	HHD 2012	HD 2012	HT 2012	HW 2012
HHD 2011	1.00							
HD 2011	0.48	1.00						
HT 2011	−0.54	−0.64	1.00					
HW 2011	0.53	0.83	−0.77	1.00				
HHD 2012	0.88**	0.48	−0.57	0.55	1.00			
HD 2012	0.45	0.91**	−0.68	0.80	0.48	1.00		
HT 2012	−0.49	−0.63	0.90**	−0.78	−0.54	−0.68	1.00	
HW 2012	0.49	0.75	−0.70	0.80**	0.52	0.78	−0.75	1.00

** Significant at P≤0.0001.

### SNP genotyping of parents and RILs

The re-sequencing of two subspecies generated two paired-end libraries with 90-bp reads, which include about 141 million raw reads in Wut and 107 million in Bre respectively (Table S1 in file S1). The whole reference genome of *B. rapa* v1.1 (Ref) contains 10 chromosomes with 256 Mb, and 40 thousand scaffolds with 27 Mb [17]. According to this standard, we deduced that the sequencing depth of the parental lines was about 45 fold in Wut and 33 fold in Bre, and the mapped depth was 23 fold in Wut and 26 fold in Bre. Raw SNPs supported by location-specific reads included 1.54 million between Wut and the reference genome, and 1.20 million between Bre and the reference genome (Table S2 in file S1). Each SNP supported by less than 4 reads were filtered out, leading to 0.92 million high-quality SNP between Wut and Ref and 0.70 million SNP between Bre and Ref. The distance between the most adjacent SNPs was 11–100 bp. SNP density is 2–3 per 1 kb in each chromosome of Bre, 3–4 per 1 kb in each chromosome of Wut. Distribution of SNP number per 10 kb along chromosomes was described in Fig. S1 in file S1. The SNPs different between Bre and Wut was 1.05 milllion.

On the basis of low-coverage resequencing (0.2×) of 150 RIL lines (Accession: SRX181272), we called SNPs by alignment with the reference genome of *B. rapa vs Chiifu-401-42* [17] in an allele-specific manner. The SNPs of RILs non-existent in the parents were filtered out. Comparison of SNPs in each RIL with those between two parents Wut and Bre (1.05 million high-quality SNPs) revealed about 0.1–0.25 million SNPs on average in each RIL line, meaning that each 100-kb interval had about 30–80 SNPs to call its genotype (Fig. S2 in file S1).

**Figure 3:**
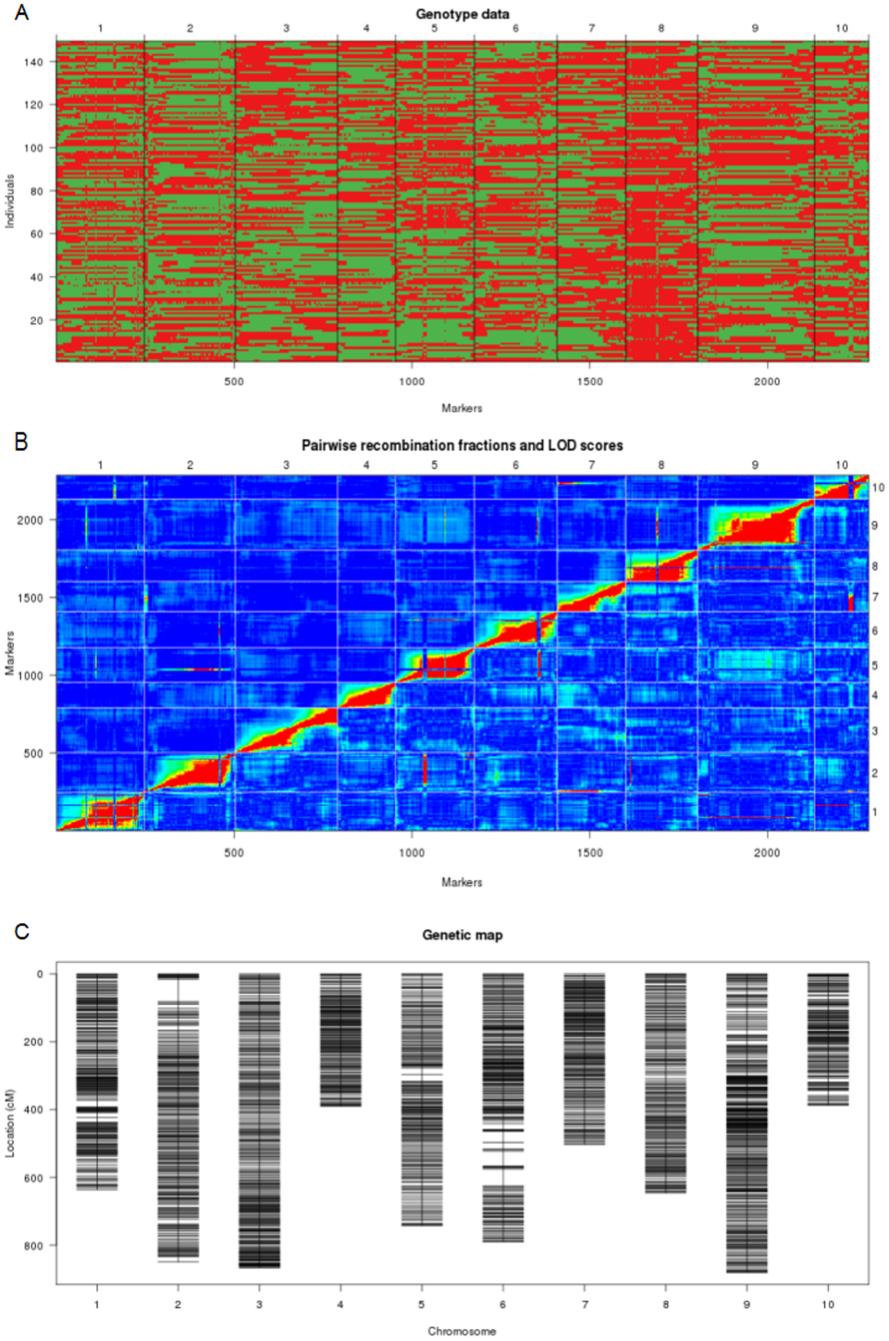
Recombination map and linkage map. (A) Recobination map of 150 RILs; (B) Pairwise recombination fractions and LOD scores; (C) Genetic linkage map after filtering markers that recombination rates higher than 20%.

In rice, the SNP data from low-coverage sequencing of RIL population have been directly applied to genotyping. Using the method of Xie et al. [9], we genotyped 150 RILs of *B. rapa* with HMM. Consecutive SNP sites with the same genotype were lumped into bins and a breakpoint was assumed at the transition between two different genotype bins. By scanning the whole genome in 150 RILs with 100-kb windows, we detected 2305 recombination events in total (Fig. 3A). The breakpoints shuffled the genome into 2315 bins, which were severed as the almost saturated non-redundant markers (genetic markers) for generation of a linkage map.

### High-quality genetic map

Using the raw recombination map, the average recombination rate between adjacent recombination bins was 2.5%. Unexpectedly, some abnormal recombination sites (recombinant hotspots) with recombination rates higher than 40% or even 50% were observed in chromosomes 1, 5, 6, 8 and 10 (Fig. 3B). The resultant linkage maps were abnormal, with some error genetic markers. Complex structural variation such as transchromosomal rearrangement or inversion may happen between two parents, or that the bug of the genome assembly may exist in the physical map. We explained that the abnormal recombination sites were caused by assembly errors in some regions of the reference genome. By deleting the recombination bins having recombinant rates higher than 20%, we obtained 2209 high-quality genetic markers (Fig. 3C).

### QTL mapping of head traits

**Figure 4:**
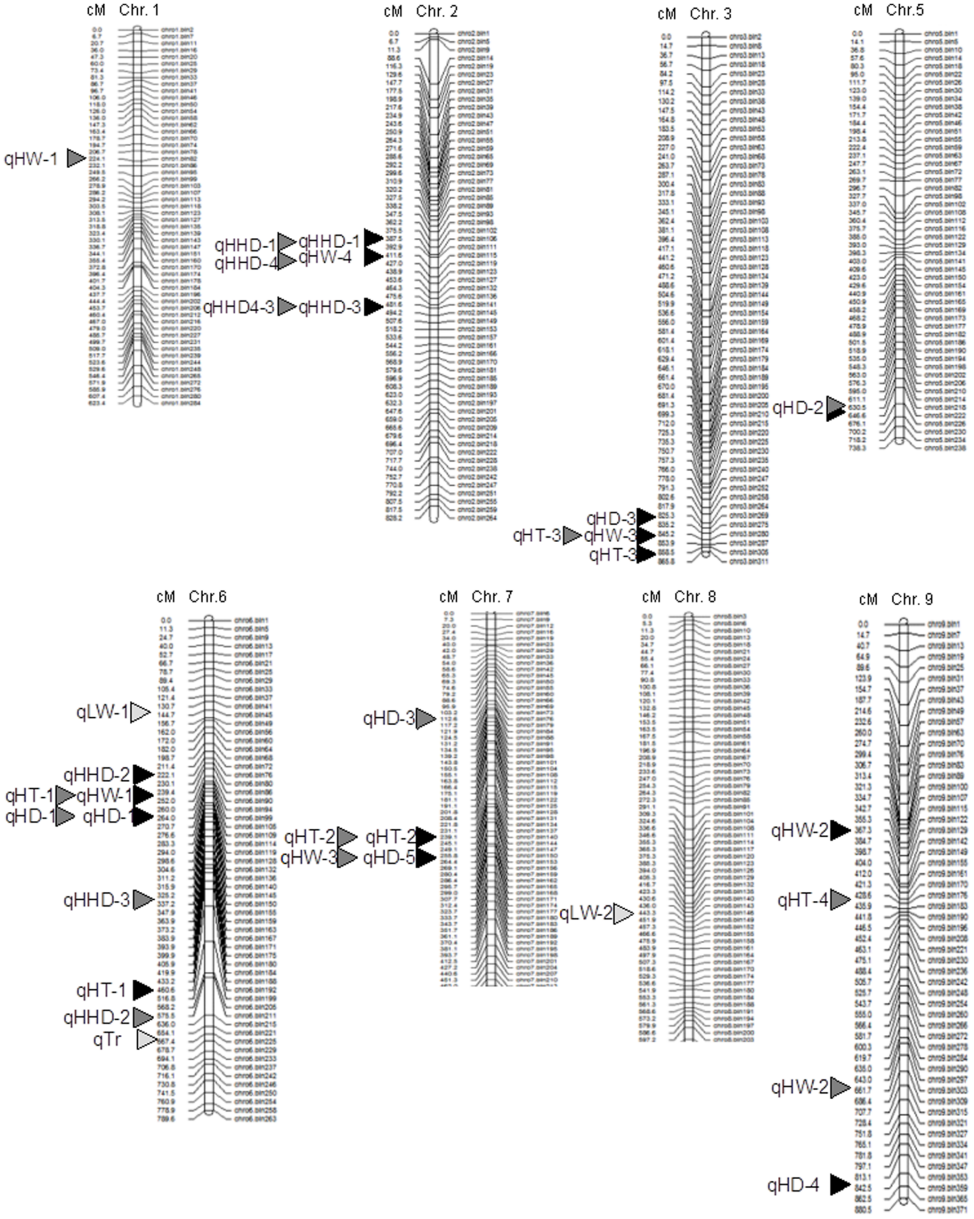
Linkage relationship between the QTLs for head traits. The names of QTLs are described in Table 3. The black and grey arrow heads indicate the locations of QTLs in 2011 and 2012, respectively. The locations of the QTLs for qTr and qLW in the two years are the same and indicated by white arrow heads.

With high-density genotype data, high-resolution linkage mapping was performed using WinQTLCart for composite interval mapping (CIM) [19]. In total, 18 trait loci associated with 6 traits were identified that satisfied the threshold LOD of more than 3 and phenotypic effect (R^2^) of more than 5% (Table 3; Fig. 4). Two QTLs for head height-to-diameter ratio (HHD) were detected repeatedly in both trails, whereas two and one QTLs for head diameter (HD) and heading time (HT) in both the trails, respectively. Some QTLs for HHD were co-located on the chromosomes with those for HD, HT or HW. For examples, qHHD-2 overlapped with qHD-1, and qHW-1, which were physically linked to each other. Similarly, qHD-5 overlapped with qHT-2. Likely, some head traits shared the same genetic loci.

**Table 3:** QTLs of 6 heading traits in two years.

Trait Loci	Chromosomes	Position (M)	LOD	R2	LOD2_L (M)	LOD2_R (M)
*2011*						
qHHD-1	2	0.383	4.993	0.168	0.276	0.613
qHHD-2	6	2.331	3.739	0.089	2.143	2.372
qHHD-3	2	4.893	3.116	0.068	4.848	4.909
qHD-1	6	2.534	13.877	0.267	2.393	2.634
qHD-2	5	6.891	5.985	0.094	6.835	7.002
qHD-3	3	8.219	5.685	0.096	8.096	8.341
qHD-4	9	8.248	4.182	0.061	8.131	8.472
qHD-5	7	2.631	3.711	0.056	2.556	2.807
qHT-1	6	4.786	15.939	0.300	4.606	4.846
qHT-2	7	2.265	10.028	0.154	2.074	2.341
qHT-3	3	8.615	6.840	0.100	8.485	8.652
qHW-1	6	2.424	12.916	0.229	2.300	2.520
qHW-2	9	3.633	5.056	0.082	3.438	3.670
qHW-3	3	8.263	4.312	0.057	8.253	8.346
qHW-4	2	4.005	4.227	0.061	3.975	4.112
*2012*						
qHHD-1	2	0.363	6.167	0.146	0.266	0.559
qHHD-2	6	5.995	4.830	0.087	5.356	6.099
qHHD-3	6	3.356	3.795	0.057	3.171	3.394
qHHD-4	2	4.826	3.036	0.052	4.809	4.849
qHD-1	6	2.530	8.389	0.182	2.392	2.634
qHD-2	5	6.891	6.048	0.100	6.853	7.002
qHD-3	7	1.079	3.357	0.067	1.032	1.126
qHD-4	2	4.015	3.283	0.065	3.975	4.062
qHT-1	6	2.334	11.043	0.195	2.306	2.434
qHT-2	7	2.321	7.239	0.126	2.131	2.381
qHT-3	3	8.329	6.569	0.075	8.233	8.339
qHT-4	9	4.349	4.140	0.057	4.326	4.385
qHW-1	1	2.241	4.800	0.107	2.181	2.358
qHW-2	9	6.590	3.774	0.085	6.570	6.640
qHW-3	7	2.671	3.630	0.074	2.644	2.691
*2011* and *2012*						
qTr	6	6.627	43.296	0.734	6.620	6.628
qLW-1	6	1.401	10.511	0.282	1.381	1.421
qLW-2	8	4.599	4.620	0.131	4.573	4.629

### Selection of candidate genes

**Figure 5:**
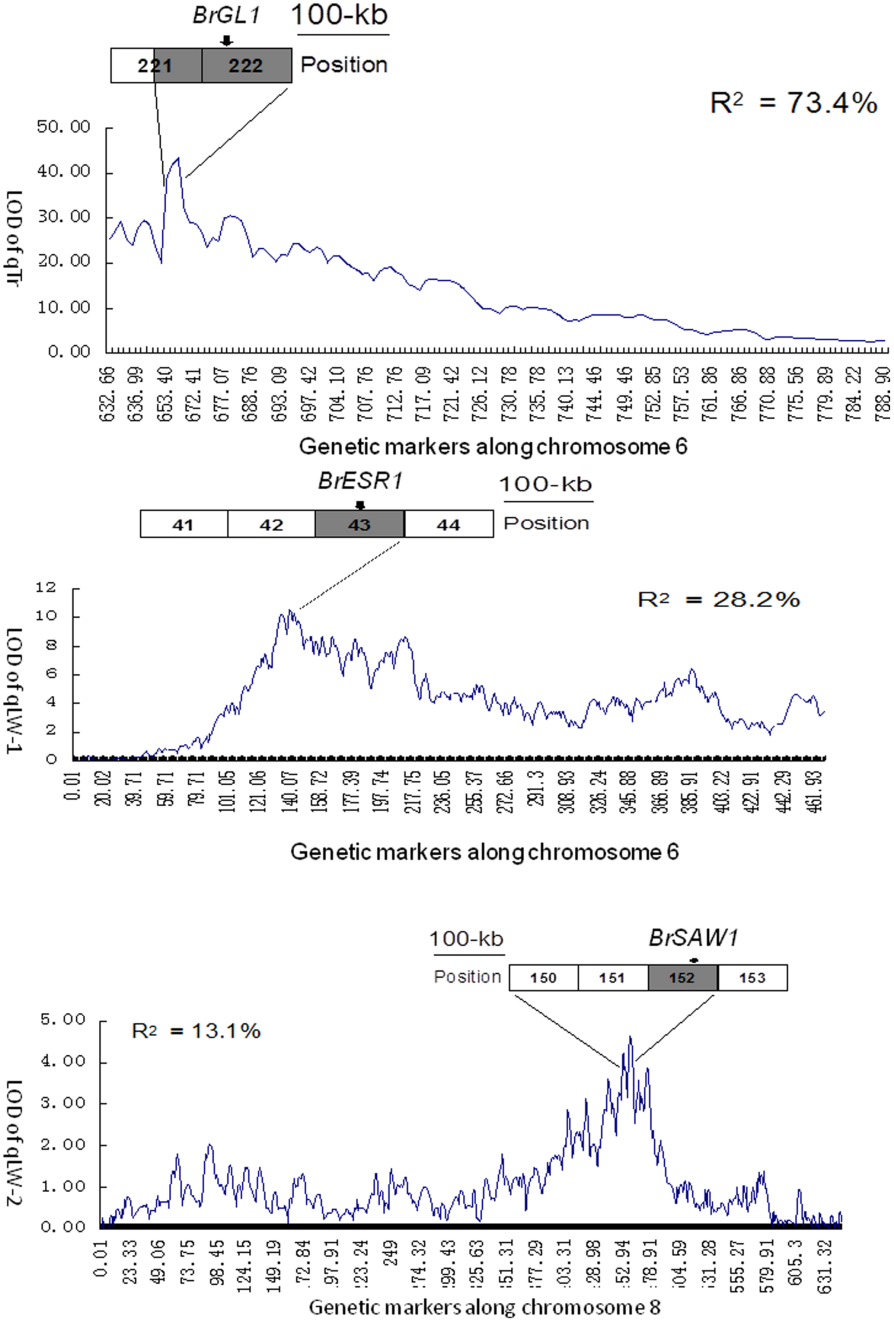
Precise locations of three QTLs and candidate genes. Curves indicate chromosomal locations and LOD values of detected QTLs. Names and phenotypic effect (R2) of the QTLs are indicated. Recombination bins near the LOD peaks of the QTL are illustrated as horizontal bars with their numbers labeled inside. Shaded bin overlaps the LOD peak of a QTL. The portion of a curve and corresponding bins defining the 95% confidence interval of a QTL are bracketed by two lines. The relative physical position of a candidate gene in the bin is indicated by an arrow head.

The close relationship between Brassica and Arabidopsis makes it possible to find some candidate genes by referring to the known research in the model plant. For selection of the potential genes associated with the phenotype, all the genes in these confident regions of trait loci were annotated with the information of Arabidopsis homologous genes and functional descriptions. In the most confident 150-kb region of qTr (*R* ^2^ = 73.4) (trichome number) on the chromosome 6, a homolog of Arabidopsis *GL1* (*GLABROUS*) located near the peak signal (Fig. 5A). *GL1* is a central regulator of trichome cell fate decision [20], and its loss-of-function mutant does not produce trichome. The qTr allele of Wut contained three nonsynonymous substitutions, compared with that of Bre. Within the population of RIL lines, most had the short or no petiole in head leaves because leaf wings were along the petioles. The gene homologous to Arabidopsis *ESR1* may be the candidate gene since it located in the 500-kb confident region, near the peak signal (Fig. 5B). In Arabidopsis, both AP2 domain transcription factors, *ESR2* and *ESR5* (*LEAFY PETIOLE*, *LEP*), have the phenotype with a shorter petiole [21], [22]. *SAW1* was found near the peak signal in the 900-kb confident region of qLW-2 (*R* ^2^ = 13.1) (Fig. 5C). In Arabidopsis, *saw1saw2* mutant displayed leaf serrations in lateral organs [23]. *BrpGL1*, *BrpESR1*, and *BrpSAW1* were the three candidate genes that may regulate development of trichome, petiole, serration, and cell division and were supposed to function in formation of leafy heads. The fine mapping has been designed to identify the QTL alleles that control more important traits such as leaf incurvature, large blade, small inclination angle, and low leaf shape index.

## Discussion

Genotype data quality of the parental lines is one of the most important elements for genotype calling in mapping population. In rice, both of the two subspecies (*indica* and *japonica*) are used as the parental lines of the mapping population with high quality SNP information [8]. In this case, the SNP data of low-coverage sequencing of the RIL population are directly applied to genotyping, without deep sequencing of biparents. In *B. rapa*, however, the reference genome sequences are incomplete [17]. Both genotypes we studied are different from the reference genotype *Chiifu-401-42*. Using the method of Huang et al. [8], we experienced segregation distortion in RIL population of *B. rapa* and double cross-over between two adjacent 100-kb intervals that were apparently caused by false SNPs. Through assistance of Hidden Markov Model in the RIL population, such false positive SNPs were filtered out.

Recombination events in RIL population with fixed-size limit the highest resolution in linkage mapping. Detecting all the recombination breakpoints in the population could obtain the saturated genotype markers. By using low coverage sequencing of RILs, we detected the SNPs that distribute in the whole genome with high density. Each 100-kb interval has 30–80 SNPs for genotype calling. The recombination rate distribution of adjacent bins in the recombination map of the RIL population displayed normal and abnormal recombination hotspots. The inconsistency of the recombination fractions and the LOD score of the genetic map suggests that complex structural variation such as transchromosomal rearrangement or inversion may happen between two parents, or that the error of the genome assembly may exist in the physical map. Abnormal or normal recombination hotspots extend the distance of the genetic map, and produce noise with false positive peak signals in QTL mapping. Identification of assembly error of the reference genome enables us to get rid of false QTLs, thus resulting in near-saturated and complete genetic linkage map.

In total, 0.9 million high-quality SNPs were detected between the non-heading Chinese cabbage (Wut) and the heading Chinese cabbage (Ref). Multiple SNPs caused nonsynonymous mutation, and especially premature termination. The SNPs identified in the whole genome between these two subspecies are useful for the development of classical molecular markers for map-based cloning in the further backcross population. On the basis of the close relationship in genomic sequences between Brassica and Arabidopsis, we identified a subset of candidate genes. Interestingly, the leaf phenotypes of some RILs were similar to those of Arabidopsis mutants of miRNA genes and miRNA-targeted genes. Probably, leaf development during heading is precisely regulated through miRNA pathways. To define the roles of miRNA-directed pathways in morphological genetics of leafy heads, we are now attempting to identify QTLs for the expression of miRNA-related genes.

How the incurved leaves are organized to form a leafy head is an interesting question. Some genetic factors should act to monitor the alleles responsible for head leaves. Incurvature, inclination angle, leaf size, and number of rosette leaves and shrinking leaves affect head shape, head compactness and head size. Some of the major alleles for compact head may be the organizers of heading. Cloning and functional analysis of the alleles determining the heading and three-dimensional variance of leaves will reveal the morphological genetics of leafy head formation and provide genetic means to optimize leaf shape desirable for high yield and leafy head quality.

## Materials and Methods

### Plant material and phenotyping

This study investigated the inbred line of heading Chinese cabbage (*B. rapa* ssp. *pekinensis* cv Bre), non-heading Chinese cabbage (*B. rapa* ssp. *chinensis* cv Wut), and the sixth generation population of recombinant inbred lines (RILs) derived from a cross between these two sub-species. All were grown in SongJiang SIPPE Agricutural Station, Shanghai, China in autumn of 2011 and 2012. Ten seeds per RIL line were sowed in green house on August 8, 2011, and on August 12, 2012, and grown at 22/18°C with 16/8 h light (night/dark). The five seedlings of four-leaves per RIL line were transplanted into the field. For each plot, every 30 cm in 30 m-long plots on rows 60 cm apart was designed. Ten seedlings of each of the parental lines were set for each experiment. One-month old seedlings were sampled for isolation of genomic DNA using CTAB methods.

Five plants from each RIL and parent grown in the field were randomly chosen for phenotyping. 6 head traits were measured in the open field. Head diameter (HD) was used to represent head size, height-to-diameter ratio (HHD) to represent head shape, fresh weight per head was calculated for head weight (HW), number of days after germination for heading time (HT). Trichome (Tr) and leaf wing (LW) were marked with the indices between 0 and 1.

### Sequencing data and alignment with reference genome

The DNA samples were sent to BGI-Shenzhen for sequencing by an Illumina Hiseq-2000 system, which produced the paired-end libraries with 2×100 bp read length. All data were submitted to The Sequence Read Archive (SRA) stores with the following accession: SRX181271 (*Brassica rapa* ssp. *Pekinensis*), SRX181266 (*B.rapa* ssp. *Chinensis*), SRX181272 (RIL population).

After cutting adapters, the mean of the quality scores and the GC proportion of raw reads were calculated. The first whole genome sequence of the Brassica A genome species (*Brassica rapa vs Chiifu-401-42*) was used as the reference (http://brassicadb.org). The raw paired-end libraries of Wut and Bre were aligned to the reference genome using SOAPalligner (SOAP2) software with the parameter “-l 32 -s 40 -v 5 -m 10 -×1000 -r 2”, as well as bwa/samtools with the default parameter [24]. The effective depth of reads was calculated as follows: the total length of raw reads (90 bp plus the number of reads) minus that of the filter reads that could not match to the reference genome, all divided by the length of the reference genome. The 150 paired-end libraries from the RILs lines were also mapped to the reference genome using SOAP2 with the same parameters.

### Identification and annotation of SNP

Based on the alignment file of SOAPalligner, the reads that aligned with the 10 different chromosomes were separated into 10 files, and ordered according to the physical location of the chromosome. SOAPsnp was used for consensus-calling and SNP detection of each chromosome using Bayesian theory [25], and the “-Q j -r 0.0005 -e 0.001 -t -u -L 90” parameter. Next, the true SNP were selected with the following criteria: (1) no second heterozygous base existed; (2) there was a quality score over 20; and (3) there were at least five supported reads. The reference coverage of re-sequencing was calculated as follows: the number of base-pairs without any supported reads divided by the number of whole genome base-pairs, and the quotient was then subtracted from one. The SOAP files of all 150 RILs were also separated into 1500 files, and the SNPs between the parents were detected in each file by comparison with the parent SNP.

Based on the alignment file of the bwa/samtools, Pindel software was used to detect the deletion and short insertion using the default parameter [26], for which the number of supported split reads containing the breakpoint was no less than 3. The genes containing SNP and short InDel were selected by comparing the location of SNP and INDEL with those of all Brassica gene models v1.1 (http://brassicadb.org). Further, SNPs were determined whether they were located in exon region, and whether they caused synonymous mutation, nonsynonymous mutation, premature termination, or abnormal termination.

### Genotyping calling

The R package MPR [9] was used to perform RIL genotype calling with a Hidden Markov Model, and recombination events were detected with a 100 kb window.

### Construction of genetic linkage map

The chromosome regions shuffled by recombination breakpoints of the whole RIL population were defined as bins, and the recombination rates (< = 50%) of ordered adjacent bins were transformed to centiMorgan (cM) using the Kosambi function. Genetic linkage maps were constructed, and recombination fractions and LOD scores, pairwise, were displayed using the R package qtl (http://www.rqtl.org). After filtering out recombination hotspots (R> = 20%), high-quality genetic map was constructed with non-redundant genetic markers.

### QTL analysis

Using the genetic map and phenotype data, QTL study was conducted with composite interval mapping method (CIM) implemented in software Windows QTL Cartographer V2.5. The CIM was using Zeng's statistics model [19]. The CIM analysis was run with forward and backward stepwise regression, a window size of 10 cM, and a step size of 1 cM. Experiment wide significance (P<0.05) thresholds for QTL detection were determined with 500 permutations. The location of a QTL was described according to its LOD peak location and the surrounding region with 95% confidence interval calculated using WinQTLCart. And QTLs that LOD above 3 and phenotype effect (R2) above 5% were selected.

QTL mapping was carried out with WinQTLCart2.5 using the composite interval mapping method (CIM) with the statistical model [19] as
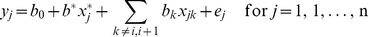
where *y_i_* is the trait value of the *j*th individual, *b*_0_ is the mean of the model, b* is the effect of the putative QTL expressed as a difference in effects between homozygote and heterozygote, x_j_* is an indicator variable, 1 or 0, with probability depending on the genotypes of markers *i* and *j* and the position being tested for the putative QTL, *b*_0_ is the partial regression coefficient of the phenotype *y* on the *k*th marker, x*_jk_* is a known coefficient for the *k*th marker in the *j*th individual, taking a value 1 or 0 depending on whether the marker type is homozygote or heterozygote, and *e_j_* is a random variable.

### Identification of candidate genes

The small regions of large effects QTL (95% confidence interval) were overlapped with the locations of the genes homologous to the Arabidopsis genes reported in the previous study. If the alleles in these small regions show nonsynonymous SNPs compared with the sequences of one parent and are consistent with the phenotypes such as trichome and leaf wing in this parent, they are regarded as the candidate alleles. Then, the parents and RILs with the candidate alleles are compared with the mutant phenotype of Arabidopsis mutants. The functions of the selected candidate genes are deduced and explained according to the reported homologs in Arabidopsis.

## Supporting Information

File S1 **Supplemental tables and figures. Figure S1, Distribution of SNPs in 10-kb intervals along 10 chromosomes of Bre and Wut. Figure S2, Sequencing depth and SNP number per 1 kb in RILs.Table S1, Overview of the resequencing data of Bre and Wut. Table S2, Total numbers of SNPs in Bre and Wut, compared with the reference genome (Ref).**
(DOC)
