# Supplementary material for: QTL Mapping of Leafy Heads by Genome Resequencing in the RIL Population of Brassica rapa
Source: PLoS One. 2013 Oct 28;8(10):e76059. doi: 10.1371/journal.pone.0076059 (PMC3810141; doi:10.1371/journal.pone.0076059)
Supplement: File S1 — Supplemental tables and figures. Figure S1, Distribution of SNPs in 10-kb intervals along 10 chromosomes of Bre and Wut. Figure S2, Sequencing depth and SNP number per 1 kb in RILs.Table S1, Overview of the resequencing data of Bre and Wut. Table S2, Total numbers of SNPs in Bre and Wut, compared with the reference genome (Ref). (DOC) [file pone.0076059.s001.doc]

Table S1. Overview of the resequencing data of Bre and Wut.

| Cultivars | Bre | Wut |
| --- | --- | --- |
| Raw reads | 141086812 | 107772750 |
| Raw base (G) | 12.7 | 9.7 |
| GC (%) | 40.6 | 36.0 |
| Mapped reads | 82518168 | 72661968 |
| Mapped percentage (%) | 58.5 | 67.4 |
| Mapped Bases (G) | 7.4 | 6.5 |
| Mapped depth | 26.2 | 23.0 |
| Coverage (%) | 85.4 | 87.2 |

Table S2. Total numbers of SNPs in Bre and Wut, compared with the reference genome (Ref).

| Genomes | Raw SNPs | High-quality SNPs |
| --- | --- | --- |
| Wut vs. Ref | 1541990 | 922951 |
| Bre vs. Ref | 1201810 | 700316 |
| Wu vs. Bre |  | 1049563^a^ |
| Shared in Wut and Bre |  | 286852 |

^a^ Raw SNPs include false positive SNPs.


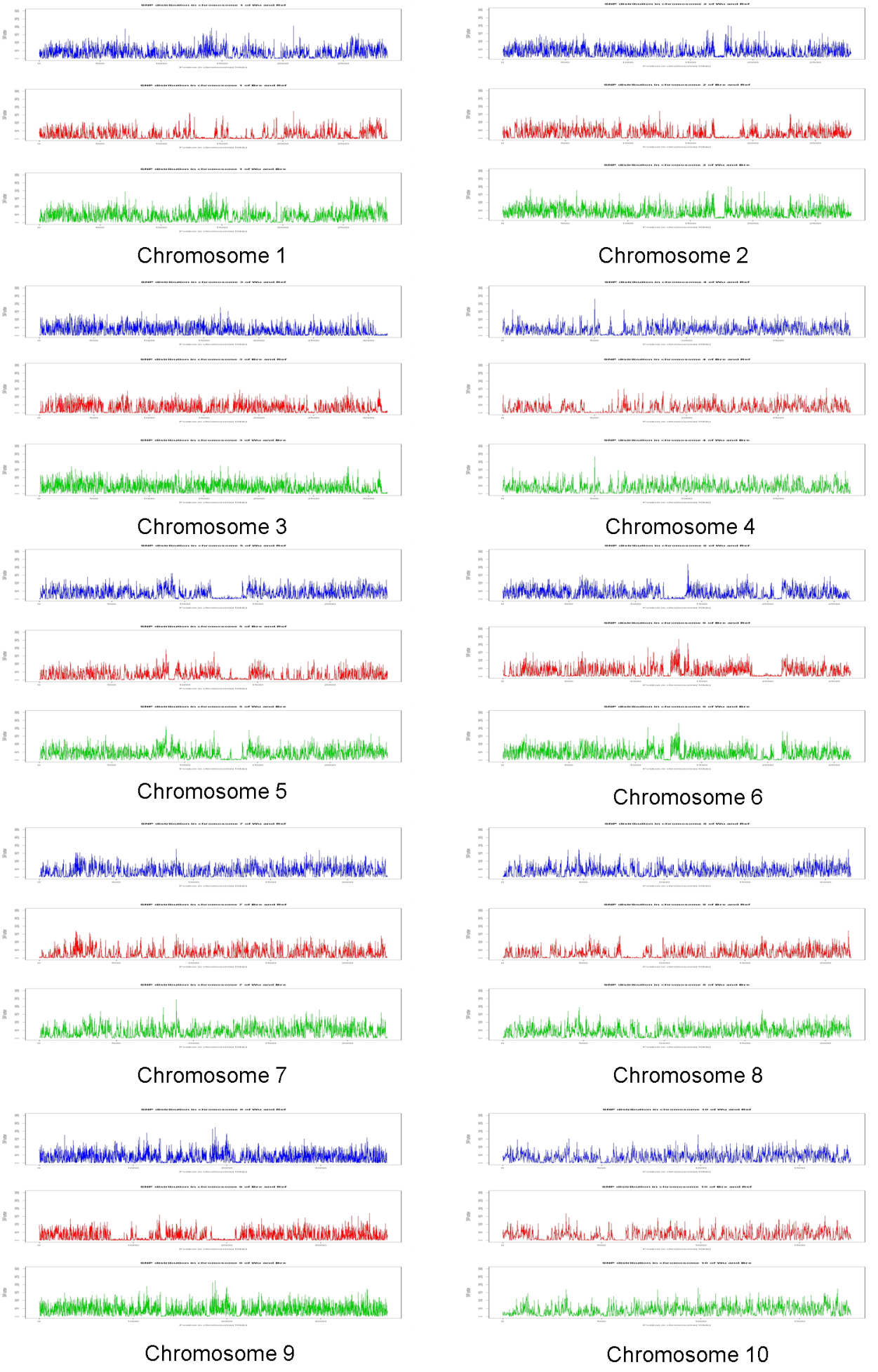


Figure S1. Distribution of SNPs in 10-kb intervals along 10 chromosomes of Bre and Wut. Red: Bre vs. Ref. Blue: Wut vs. Ref. Green: Bre vs. Wut.


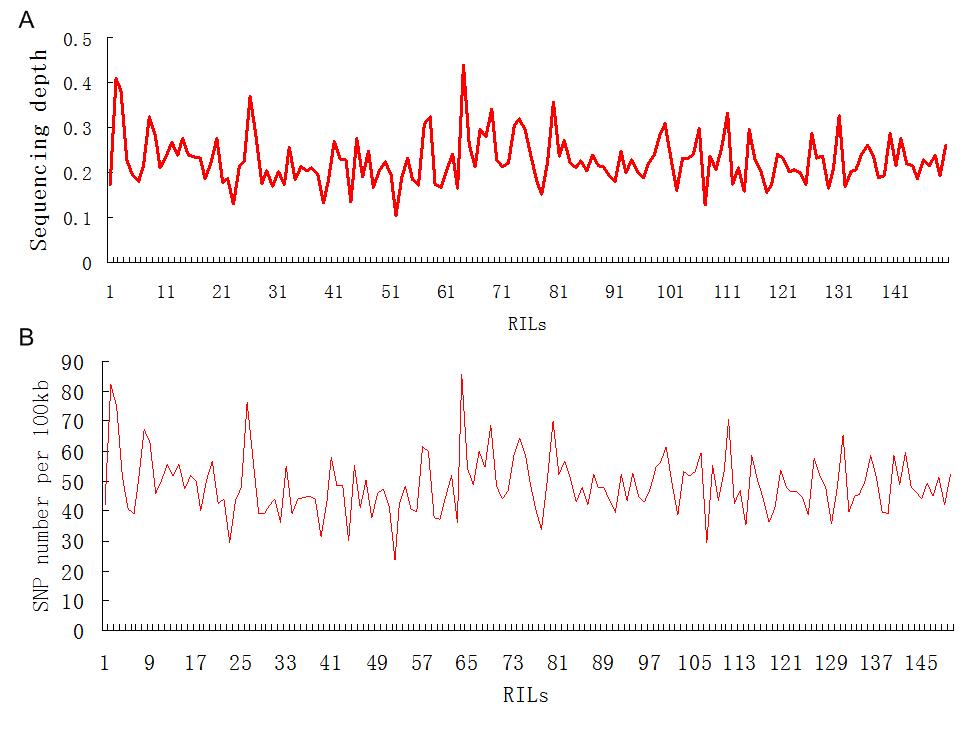


Figure S2. Sequencing depth and number of SNPs per 1 kb in RILs
